# A new Late Devonian genus with seed plant affinities

**DOI:** 10.1186/s12862-015-0292-6

**Published:** 2015-02-26

**Authors:** Deming Wang, Le Liu

**Affiliations:** Department of Geology, Key Laboratory of Orogenic Belts and Crustal Evolution, Peking University, Beijing, 100871 China

**Keywords:** China, Early seed plant, Famennian, Late devonian, Leaf trace, Petiole, Protostele, Tizikou formation, *Yiduxylon trilobum*

## Abstract

**Background:**

Many ovules of Late Devonian (Famennian) seed plants have been well studied. However, because few taxa occur with anatomically preserved stems and/or petioles, the vascular system of these earliest spermatophytes is little understood and available data come mostly from Euramerica. There remains great controversy over the anatomical differentiation of Late Devonian and Carboniferous seed plant groups of Buteoxylonales, Calamopityales and Lyginopteridales. Protostele evolution of these early spermatophytes needs more research.

**Results:**

A new taxon *Yiduxylon trilobum* gen. et sp. nov. with seed plant affinities has been discovered in the Upper Devonian (Famennian) Tizikou Formation of Hubei Province, China. It is represented by stems, helically arranged and bifurcate fronds with two orders of pinnae and planate pinnules. Both secondary pinnae and pinnules are borne alternately. Stems contain a small protostele with three primary xylem ribs possessing a single peripheral protoxylem strand. Thick secondary xylem displays multiseriate bordered pitting on the tangential and radial walls of the tracheids, and has biseriate to multiseriate and high rays. A narrow cortex consists of inner cortex without sclerotic nests and sparganum-type outer cortex with peripheral bands of vertically aligned sclerenchyma cells. Two leaf traces successively arise tangentially from each primary xylem rib and they divide once to produce four circular-oval traces in the stem cortex. Four vascular bundles occur in two C-shaped groups at each petiole base with ground tissue and peripheral bands of sclerenchyma cells.

**Conclusions:**

*Yiduxylon* justifies the assignment to a new genus mainly because of the protostele with protoxylem strands only near the periphery of primary xylem ribs, leaf trace origination and petiolar vascular supply structure. It shares many definitive characters with Calamopityales and Lyginopteridales, further underscoring the anatomical similarities among early seed plants. The primary vascular system, pycnoxylic-manoxylic secondary xylem with bordered pits on both tangential and radial walls of a tracheid and leaf trace divergence of *Yiduxylon* suggest transitional features between the early spermatophytes and ancestral aneurophyte progymnosperms.

**Electronic supplementary material:**

The online version of this article (doi:10.1186/s12862-015-0292-6) contains supplementary material, which is available to authorized users.

## Background

Seed plants (spermatophytes) began to diversify in the Famennian (374–359 million years ago) of the Late Devonian and many fossils have been documented since the Carboniferous [1]. Till now, about twenty Famennian genera with ovules and/or pollen organs have been studied in detail [2]. Most of these earliest seed plants were collected from Laurussia (Europe and North America) and only two genera from China, i.e., *Kongshania* [3] and *Cosmosperma* [2]. However, few plants have been recognized on the basis of stem and/or petiole anatomy. In contrast, there are more than forty Early Carboniferous stem morphotaxa throughout Euramerica [4]. Such taxa may be the primary line of evidence for phylogenetic analyses of early spermatophytes [5].

Famennian seed plants known for their stem vascular system include *Buteoxylon* [6], *Kerryoxylon* [7], *Laceya* [8,9], *Tristichia* [7] and cf. *Tetrastichia bupatides* [7] from Ireland, and *Elkinsia* from USA [10]. They are (tentatively) attributed to the Buteoxylonales (*Buteoxylon*), Elkinsiales (*Elkinsia*) and Lyginopteridales (*Kerryoxylon*, *Laceya*, *Tristichia* and *Tetrastichia*). Anatomically preserved spermatophytes from the New Albany Shale of USA have raised great controversy over their Latest Devonian or earliest Carboniferous age [11-19].

Buteoxylonales, Calamopityales and Lyginopteridales (or Buteoxylonaceae, Calamopityaceae and Lyginopteridaceae) are represented by numerous stem morphotaxa and/or fertile organs from the Late Devonian and especially Carboniferous. However, as noted by many workers such as [4,20], the definition and relationship of these three orders need reinterpretation because of shared characters and the lack of whole plant knowledge. These early spermatophytes have been interpreted as lianas or upright plants [20,21], and their possession of sclerotic nests in the stem inner cortex has been interpreted as a response to insect infections [15,20]. The derivation of the early spermatophyte eustele from protostele has been widely accepted [1,22,23], but the evolution of the spermatophyte protostele from the ancestral aneurophyte progymnosperm protostele is poorly understood.

South China was an isolated plate in the Late Devonian and showed great diversification of many plant lineages excluding seed plants. This paper now reports a new genus with seed plant affinities, *Yiduxylon trilobum* gen. et sp. nov., having both vegetative morphology and stelar structure including petiolar vascular supply preserved from the Upper Devonian (Famennian) Tizikou Formation of Hubei Province, South China. The age of this formation is determined mainly by the stratigraphic sequence and comparisons of the spore assemblages. Based on morphological and anatomical studies of stem/frond as well as on detailed comparisons, this paper discusses the taxonomy and growth habit of *Yiduxylon*, and the evolution of seed plant protostelic architecture during the Late Devonian and earliest Carboniferous. The stelar feature and leaf trace production of *Yiduxylon* indicate transitional anatomical traits in the evolution of the seed plants from the Middle to Late Devonian aneurophytes, although the latter group is currently unknown in South China.

## Material and methods

Fossils were collected from the Upper Devonian Xiejingsi (Hsiehchingssu) Formation sensu lato, on a hill named Tizikou, which is to the south of Maohutang village, Wangjiafan Town, Yidu City, western Hubei Province, China. In the Three Gorges area of the Yangtze River including western Hubei, the Upper Devonian strata are referred to the Huangjiadeng Formation and overlying Xiejingsi Formation sensu lato (see summary in [24]). The latter formation includes the Frasnian-Famennian Xiejingsi Formation sensu stricto, Famennian Tizikou Formation and Changyang Formation in ascending order [25]. The Changyang Formation containing marine invertebrates is Famennian [25,26] or Lower Carboniferous (basal Tournaisian) [27,28]. The present plant occurs together with a lycopsid *Sublepidodendron songziense*. The latter species has been discovered and studied in detail from the Famennian Tizikou Formation of Songzi City, western Hubei and from the Famennian Wutong (Wutung) Formation of Chaohu City, Anhui Province [24,29]. Miospore assemblages indicate that the Tizikou Formation in western Hubei and northwestern Hunan is similar in age to the Famennian (Fa2d-Tn1a) Xikuangshan Formation (upper part) and Shaodong Formation (lower part) in central Hunan, the Zhewang Formation and Gelaohe Formation (lower part) in southeastern Guizhou, Wutong Formation (upper part: Leigutai Member) in the lower reaches of the Yangtze River, and the Wangjiadian Formation in southern Gansu [30-33]. Some spores in the PN assemblage, e.g., *Vallatisporites pusillites*, *Aneurospora greggsii* and *Rugospora flexuosa* occur only in Famennian deposits [33]. These data suggest that the Tizikou Formation is middle to late Famennian but not latest Famennian.

At the Tizikou section, the Tizikou Formation is about 20 m thick and characterized by black-gray mudstone and siltstone, and gray or gray-white fine sandstone [27]. There are some lycopsids and the sphenopsids *Hamatophyton verticillatum* and *H. yiduense* in the mid-lower part of this formation [27]. *H. verticillatum* and *H. yiduense* have been emended as *Rotafolia songziensis* [34], which was later anatomically studied [35]. Near the horizon of *R. songziensis*, *Yiduxylon trilobum* gen. et sp. nov. occurs as impressions and compressions in black-gray silty mudstone.

The poorly preserved stems, branches and leaves show little contrast with the rock matrix. Under a microscope, clastic material was removed by steel needles to expose the plant morphology. Coalified material of *Yiduxylon trilobum* has been well preserved, which was embedded, sectioned and ground to show the anatomy. Altogether nine stems were transversely sectioned to produce 62 slides, and six stems were longitudinally sectioned to produce 16 slides. These sections were observed with light microscope (LM). Some stems were examined with a scanning electron microscope (SEM).

We have not carried out our field works in private areas, since in China all the land belongs to the country. And we have not caused any destruction to National Nature Reserves during fossil excavation. All the fossil collecting works have been done obeying the Chinese fossil collection and mining laws, and have been allowed by local government.

## Results

### Vegetative morphology

**Figure 1:**
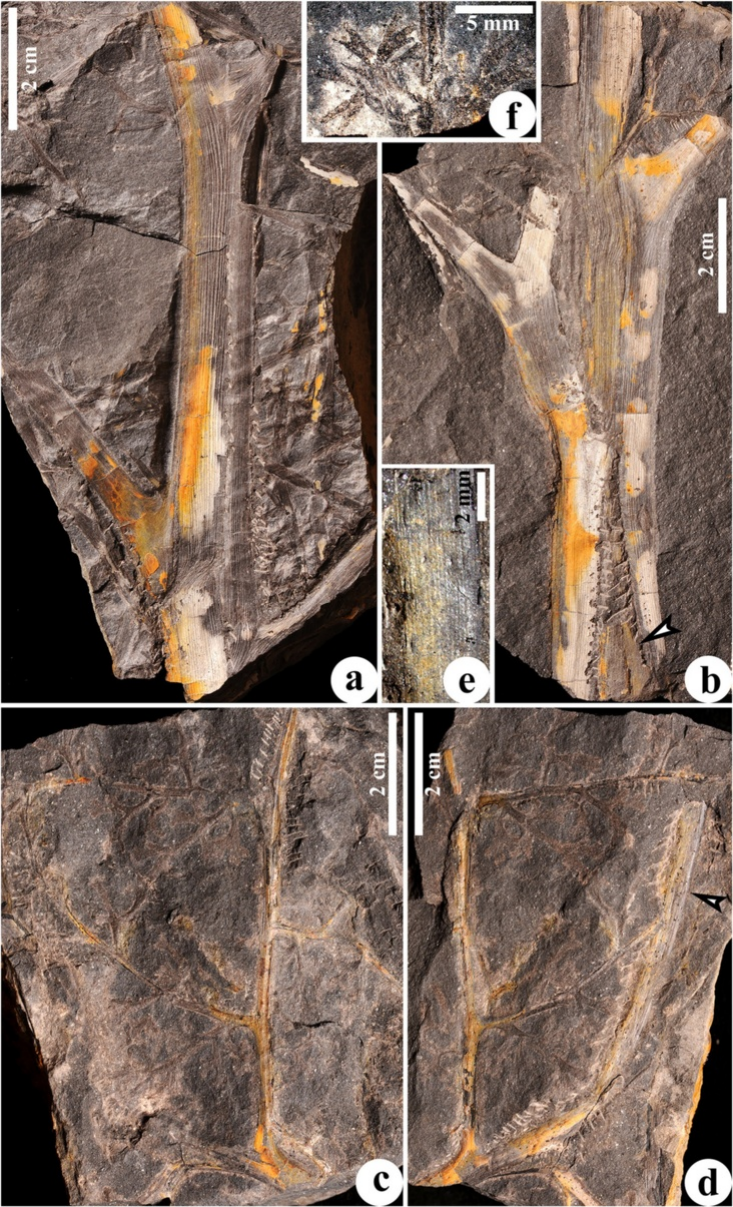
**Vegetative morphology of** ***Yiduxylon trilobum*** **gen. et sp. nov. from Hubei.** **(a)** Stem attached by three fronds. PKUB14401. **(b)** Stem with three fronds in helix, with middle frond bifurcating and lower frond extending into rock (arrow). PKUB144403. **(c-d)** Part and counterpart of specimen showing connection of frond, primary and secondary pinnae and planate pinnules in one plane. Arrow in **(d)** indicating frond rachis enlarged in **(e)**. PKUB14402a, PKUB14402b. **(e)** Enlargement of **(d, arrow)** displaying longitudinal ridges and grooves. **(f)** Secondary pinna rachis with planate pinnules. PKUB14407.

Two stems are up to 120 mm long and measure 12–20 mm in diameter (Figure 1a,b). They decrease in diameter acropetally. The proximal bifurcation of the fronds is sometimes visible at an angle of 60° (Figure 1b, middle frond). Rachises of fronds depart from the stem at 45-60°, are up to 60 mm long, and are separately 5.2-10 mm and 5.0-6.3 mm in diameter before and after proximal bifurcation. One stem has three fronds appearing to be alternately arranged (Figure 1a). In another stem (Figure 1b), the upper frond departs at 50°, the middle frond emerges at 20°, while the basal frond (Figure 1b, arrow) plunges into the rock matrix. In this case, the three fronds on this stem are borne helically, approaching a 1/3 phyllotaxis. The interval between the attachment of two adjacent frond rachises is 20–70 mm. Narrow and longitudinally parallel ridges 120–350 μm in width occur on the surface of stems and frond rachises and alternate with narrow grooves. These ridges correspond to the bands of sclerenchyma cells in the outer cortex (see below).

The part and counterpart of a specimen display an axis with lateral branches bearing planate pinnules (Figure 1c, d, Additional file 1: Figure S1a, b). This axis is about 85 mm long and 4.8-5.2 mm in diameter, and possesses longitudinal ridges (Figure 1d, arrow, e). The diameter and striations suggest that this axis may represent the upper part of a frond rachis after initial bifurcation. In this case, there are two orders of pinnae within the frond. Since plant shows little contrast with the rock matrix, the configuration of the pinnules is very difficult to recognize. However, narrow longitudinal ridges are visible on the rachises of the primary pinnae. The rachises of two primary pinnae are 1.8-2.6 mm in diameter, and attached separately at 70° and 30° to the frond rachis. One primary pinna is only basally preserved, whereas another is 76 mm long and bears four secondary pinnae. The rachises of secondary pinnae, up to 60 mm long and 0.8-1.3 mm in width, are alternately arranged. Except for the basal one, the other three secondary pinnae depart at about 90°. The distance between the attachment of two neighboring secondary pinnae is 16–21 mm. Nonlaminate pinnules are borne at 45-90° and alternately on the secondary pinna rachis, and are planate and highly dissected. A pinna rachis possesses at least seven pinnules. The distance between two adjacent pinnules is 4.6-13 mm. Pinnules are up to 21.7 mm long and about 19.4 mm wide. Each pinnule unequally dichotomizes to produce four or more units. These units, which are up to 11 mm long and about 12 mm wide, arise at 20-90°, and divide unequally twice or thrice to form narrow segments which are 0.5-1.2 mm wide (Figure 1f, Additional file 1: Figure S1).

### Anatomy

#### Stems

**Figure 2:**
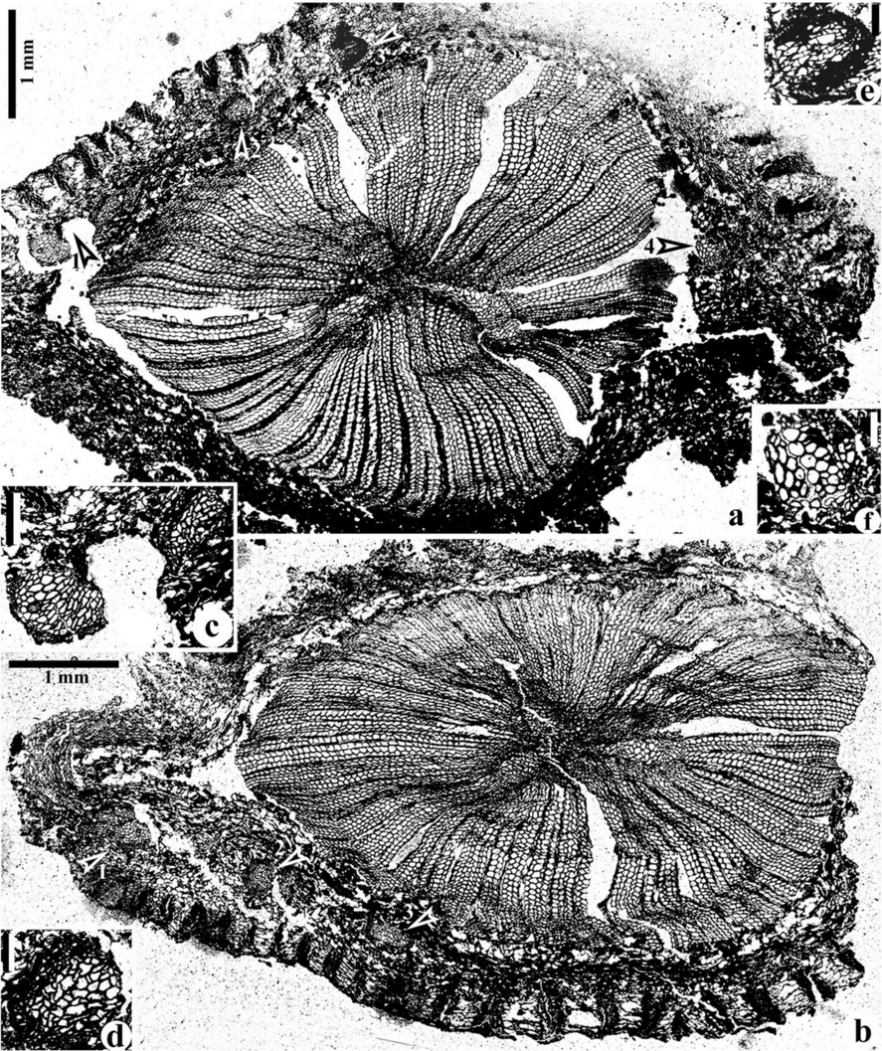
**Transverse sections of stems of** ***Yiduxylon trilobum*** **gen. et sp. nov. from Hubei.** **(a)** Enlargement of Additional file 2: Figure S2b. Arrows 1–4 indicating parts enlarged in **(c-f)**, respectively. **(b)** Stem with three primary xylem ribs, broad secondary xylem, and narrow sparganum-type cortex. Arrows 1–3 indicating parts enlarged in Figure **3** **d-f**, respectively. HY3-4. **(c-f)** Enlargement of arrows 1–4 in **(a)**, respectively. Leaf traces in inner cortex. Scale bars = 0.1 mm **(d-f)**, 0.2 mm **(c)**.

**Figure 3:**
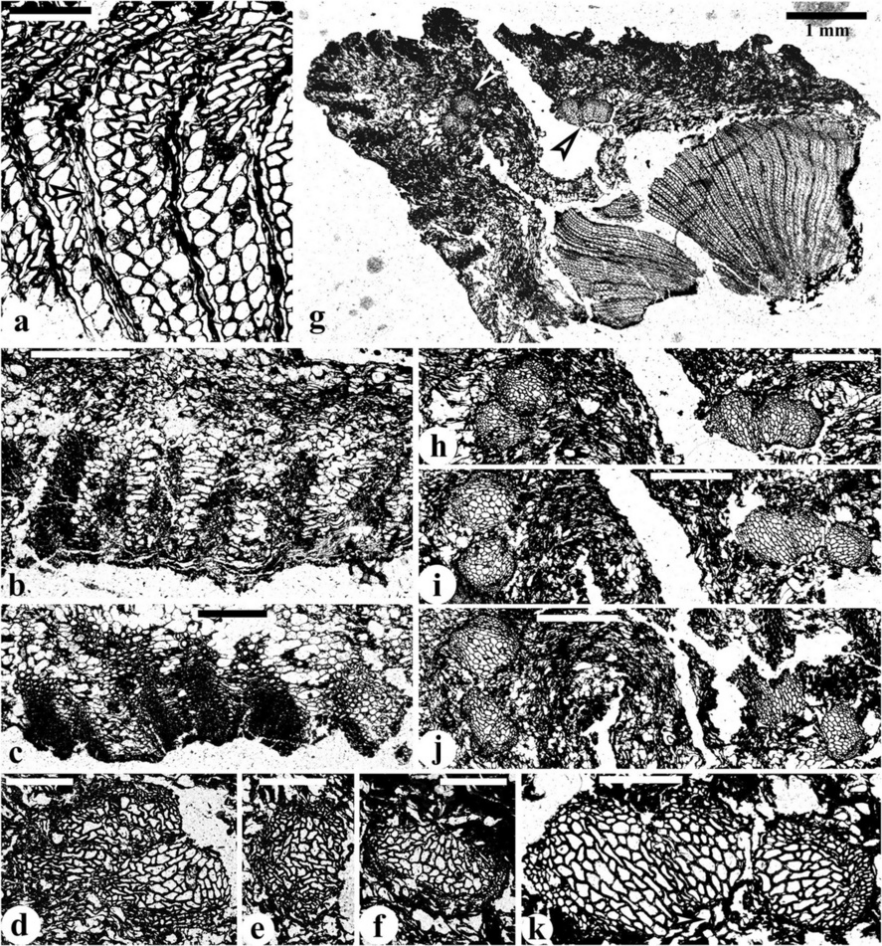
**Transverse sections of stems of** ***Yiduxylon trilobum*** **gen. et sp. nov. from Hubei.** **(a)** Secondary xylem with ray cells (arrow) and tracheids. HY6-3. **(b-c)** Inner cortex with parenchyma cells, and outer cortex with bands of sclerenchyma cells and intervening parenchyma cells. HY8-5, HY1-7. **(d-f)** Enlargement of Figure 2b (arrows 1–3), respectively, showing tangentially dividing leaf trace **(d)** and individual leaf traces in inner cortex. **(g)** Partial stem showing two dividing leaf traces in inner cortex (arrows), which are enlarged in **(h)**. HY6-7. **(h-j)** Serial transverse sections of two leaf traces showing their gradual tangential division to produce four traces. **(h)** Enlargement of **(g, arrows)**. HY6-7, HBY6-5, HBY6-3. **(k)** Enlargement of right leaf traces in **(i)**. Scale bars = 0.2 mm **(a, d-f, k)**, 0.5 mm **(b, c, h-j)**, 1 mm **(g)**.

**Figure 4:**
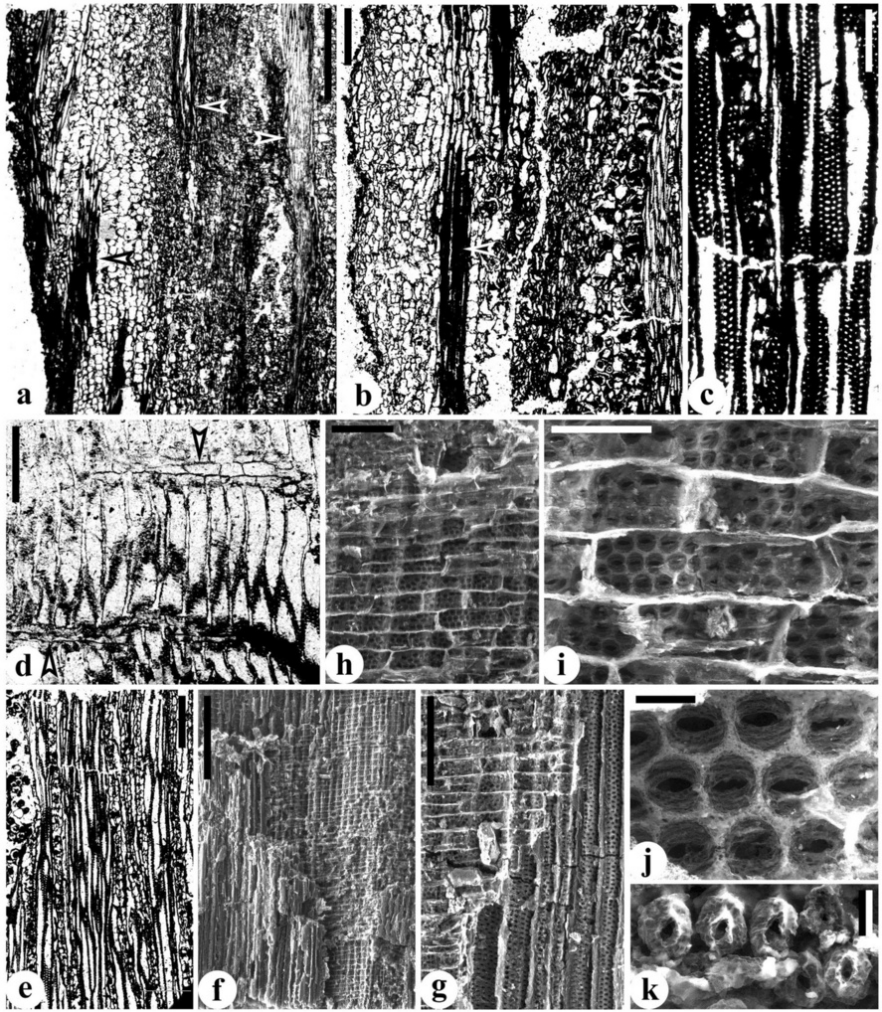
**Longitudinal view of stems of** ***Yiduxylon trilobum*** **gen. et sp. nov. from Hubei.** **(a)** Tangential section showing leaf traces (upper two arrows), sclerenchyma cells (lowermost arrow) and parenchyma cells of cortex. HY5-2. **(b)** Tangential section showing sclerenchyma (arrow) and parenchyma cells of cortex, and secondary xylem (far right). HY2-3. **(c)** Tangential section of secondary xylem showing multiseriate pitting on tangential wall of tracheids. HY7-2. **(d)** Radial section of secondary xylem showing rays (arrows) and pitting on radial walls of tracheids. HY4-3. **(e)** Tangential section of secondary xylem showing high and mainly biseriate rays. Note pitting on tangential walls of tracheids. HY9-2. **(f-k)** SEM observation of secondary xylem. **(f)** Secondary xylem in radial fracture showing very high rays. **(g)** Radial fracture showing rays and tracheids. Note pitting on radial walls of tracheids. **(h)** Radial fracture showing rays and underlying tracheids. **(i)** Enlargement of **(h)** showing ray cells and tracheids with pits. **(j)** Enlargement of **(i)** showing bordered pits of a tracheid. **(k)** Bordered pits of a tracheid. Scale bars = 1 mm **(a)**, 0.5 mm **(f)**, 0.2 mm **(b, d, e, g)**, 0.1 mm **(c, h)**, 50 μm **(i)**, 10 μm **(j, k)**.

Transverse sections of stems show a small three-ribbed protostele (actinostele), broad secondary xylem, narrow cortex and circular-oval leaf traces (Figures 2 and 3, Additional file 2: Figure S2 and Additional file 3: Figure S3). Longitudinal sections of stems demonstrate the secondary xylem and cortex (Figure 4a-e), with the former tissue being also observed with SEM (Figure 4f-k). The stems are 7.2-10.5 mm in diameter and the primary xylem is 1.8-2.0 mm in diameter (Figure 2a, b, Additional file 2: Figure S2a-d and Additional file 3: Figure S3a, b, e-g). The ratio of stelar primary xylem diameter to total stem diameter is around 1/5 (calculated from transverse section illustrated in Additional file 2: Figure S2c). The three ribs of primary xylem, which differ in length (Additional file 3: Figure S3a, arrows 1-3), are 0.5-1.2 mm long and 0.2-0.3 mm wide at their tips. The ribs become narrower toward the stelar center, being no more than 0.1-0.2 mm. The angles between two adjacent ribs of a stele are approximately 90°, 125° and 145°. The mesarch primary xylem only with tracheids (Additional file 3: Figure S3 and Additional file 4: Figure S4) possesses a single protoxylem strand located near the periphery of each rib (Additional file 3: Figures S3c, arrow, d, left arrow, h, i, and S4). The xylem rib is longitudinally continuous (Additional file 2: Figure S2a-d, Additional file 3: Figure S3e-g, Additional file 5: Figure S5a-g and Additional file 6: Figure S6a-g). Protoxylem and metaxylem tracheids are 3.0-10 μm and 13–85 μm in diameter, respectively.

Arising close to the stelar center, the secondary xylem extends outward up to a distance of 2.7 mm (Figure 2a, b, Additional file 2: Figure S2a-d). In transverse section, the secondary xylem tracheids are of various shapes (circular, oval, square, rectangular and irregular) as in the metaxylem tracheids, and are 12–75 μm in diameter and arranged in radial files. Multiseriate pits of circular and oval shape occur on both tangential (Figure 4c, e) and radial (Figure 4d, f-i) walls of secondary xylem tracheids. These pits are bordered (Figure 4j, k) and 3.0-13 μm in diameter. Rays occur between every two to about ten rows of secondary xylem tracheids (Figures 2a, b, 3a, g and 4e). Rays are usually 2 and sometimes up to 6 cells wide (Figure 4e, Additional file 3: Figure S3a, arrow), and more than 120 cells high (Figure 4e, f). Ray parenchyma cells are 65–128 μm in radial and 10–32 μm in tangential dimensions, and 22–48 μm high (Figures 3a and 4d, arrow, e-i).

The cortex sometimes appears to have been compressed to present a wing-like outline (Figure 2b, Additional file 2: Figure S2a, b). It is 0.8-1.1 mm thick (Figure 2b, Additional file 2: Figure S2a-d) and lacks sclerotic nests (Figure 3b, c). The inner cortex consists of circular and oval parenchyma cells which are 25–160 μm in diameter. Longitudinal sections show that these cells are up to 180 μm high (Figure 4a, b). Sparganum-type outer cortex contains many radial bands of sclerenchyma cells with thick walls (Figures 2a, b and 3b, c, g). These bands at the periphery of the cortex are 300–700 μm in radial and 100–520 μm in tangential dimensions, and the circular to square sclerenchyma cells range from 14–68 μm in diameter. In longitudinal sections, the sclerenchyma cells are continuous, up to 1.0 mm high and do not anastomose (Figure 4a, lowermost arrow, b, arrow). Intervening parenchyma cells are tangentially elongate between two adjacent bands with intervals of 130–270 μm.

Individual leaf traces are circular and slightly oval in outline, and their diameter is 0.3-0.4 mm near the tip of the primary xylem rib (Additional file 2: Figure S2i, k and Additional file 5: Figure S5d), but becomes 0.4-0.5 mm in the secondary xylem (Additional file 2: Figure S2c, right arrow, d, lower arrow, h, j and S5a-f), and reaches 0.3-0.7 mm in the inner cortex of the stem (Figures 2a, arrows 1–4, b, arrows 1–3, c-f and 3d-f, g, arrows, h-k, Additional file 2: Figure S2a, arrow, c, left arrow, d, upper arrow, e-g). The tracheids of the leaf traces are up to 56 μm in diameter.

Serial transverse sections of a stem indicate that each rib of the primary xylem successively initiates two leaf traces. The first trace is located at the periphery of the inner cortex (Additional file 2: Figure S2a, arrow, e and Additional file 6: Figure S6h). The tip of a xylem rib obliquely and radially expands to form a protoxylem strand representing the incipience of another leaf trace (Additional file 3: Figure S3a, arrow 3, b, d, right arrow). Then, this trace appears to tangentially diverge from the rib tip (Additional file 2: Figure S2i) and afterwards becomes detached within the secondary xylem (Additional file 2: Figure S2c, right arrow, h and Additional file 6: Figure S6i). More distally, a leaf trace diverges from the tip of another rib with protoxylem strand (Additional file 2: Figure S2k, arrow) and is then surrounded by the secondary xylem (Additional file 2: Figure S2d, lower arrow, j). This trace contains two protoxylem strands (Additional file 2: Figure S2j, arrows, k). Another trace thought to depart from the same rib tip is however invisible. It is suggested that two traces may subsequently diverge from the tip of the third rib, thus indicating a 1/3 phyllotaxy.

Serial transverse sections of another stem clearly show the successive origination of two leaf traces. One trace in the secondary xylem has been tangentially produced from a tip of the primary xylem rib (Additional file 5: Figure S5a and Additional file 6: Figure S6a), then gradually moves toward (Additional file 5: Figure S5b, c and Additional file 6: Figure S6b, c) and reaches the periphery of the inner cortex (Additional file 5: Figure S5d-g and Additional file 6: Figure S6d-g). Here (Additional file 5: Figure S5g, arrow) the trace with two protoxylem strands is tangentially elongate (Additional file 5: Figure S5i, arrows), thus indicating further division. The same rib tip obliquely and radially expands to produce a protoxylem strand representing the incipience of another trace (Additional file 5: Figure S5a-c and Additional file 6: S6a-c), which radially diverges, then passes through the secondary xylem (Additional file 5: Figure S5d-f and Additional file 6: Figure S6d-f) and reaches the inner cortex (Additional file 5: Figure S5g and Additional file 6: S6g). Because of the irregular configuration of ribbed primary xylem, the apparent radial emission of this leaf trace may result from preservation and distortion of the specimen due to compression. This trace in the secondary xylem (Additional file 5: Figure S5f, arrow) displays one protoxylem strand (Additional file 5: Figure S5h, arrow).

In the inner cortex of one stem, two leaf traces derived from a primary xylem rib begin to tangentially divide once (Figure 3g, arrows). The subsequent gradual divisions result in four traces (Figure 3h-k). Four leaf traces from a primary xylem rib occur in the inner cortex of the second stem (Figure 2a, arrows 1–3, c-e), with two traces having just tangentially separated (Figure 2a, arrow 1, c). Four leaf traces from a primary xylem rib are present in the inner cortex of the third stem (Figures 2b, arrows 1–3 and 3d-f), with two traces beginning to separate (Figures 2b, arrow 1 and 3d). Leaf traces in the cortex of these three stems have mesarch primary xylem (two protoxylem strands, see Figures 2f and 3e, k, Additional file 2: Figure S2e).

### Frond base with trace

**Figure 5:**
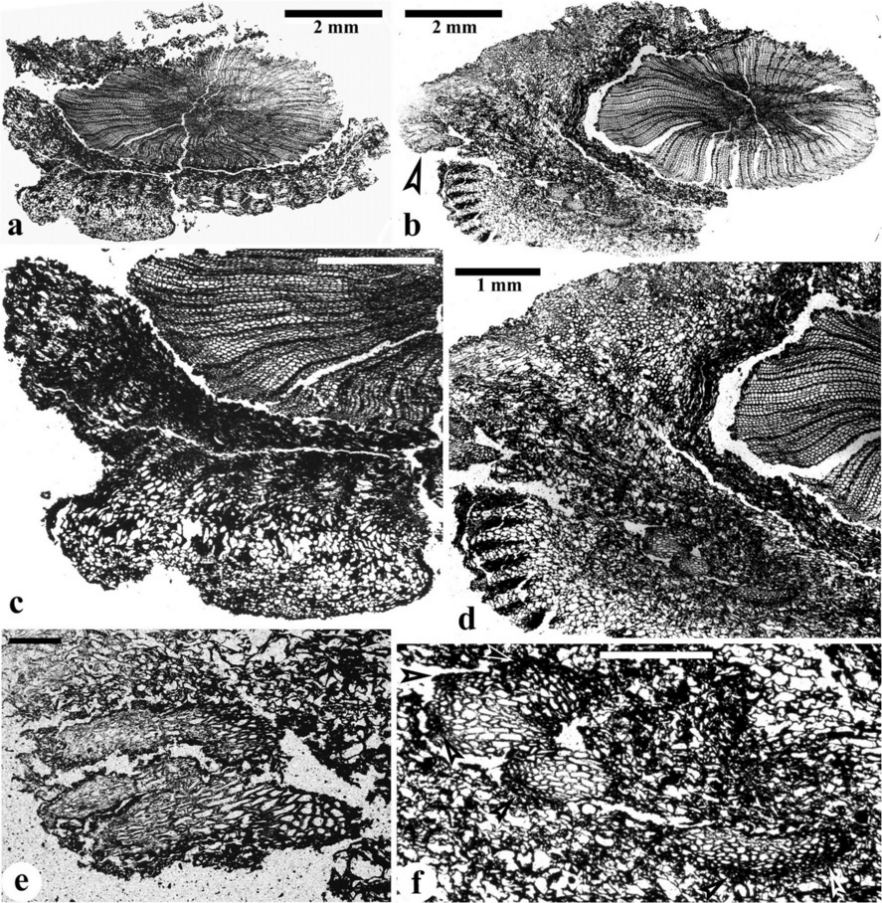
***Yiduxylon trilobum*** **gen. et sp. nov. from Hubeiv.** **(a-b)** Successive transverse sections of a stem bearing a petiole base. Stem with three-ribbed primary xylem, secondary xylem and cortex. Sparganum-type outer cortex in **(a)**. **(b)** Petiole base with vascular bundles, ground tissue and sparganum-type outer cortex. Arrow indicating part enlarged in **(e)**. Slides 4–1 and 4–6. **(c-d)** Enlargement of **(a)** and **(b)**, respectively. **(e)** Enlargement of **(b, arrow)** showing two vascular bundles in oblique longitudinal view. **(f)** Enlargement of lower left part of **(b)** showing two vascular bundles in oblique transverse view. Arrows indicating protoxylem strands. Scale bars = 1 mm **(c)**, 0.2 mm **(e)**, 0.5 mm **(f)**.

Two successive transverse sections of a stem demonstrate the attachment of a petiole base (Figure 5a-d). This stem has the same structure as the other stems, which includes a three-ribbed primary xylem of mesarch maturation, thick secondary xylem and narrow cortex with sclerenchyma cells (such cells are present only in the first section as shown by Figure 5a, b). The petiole base is ca. 5.6 mm tangentially (Figure 5b). The vascular supply of the petiole consists of four bundles, with two shown in oblique longitudinal section (Figure 5b, arrow, e) and two in oblique transverse section (Figure 5f). The latter two bundles are tangentially oriented, asymmetrical and C- or broadly U-shaped (adaxially concave). They are ca. 2.0 mm tangentially and 0.2-0.4 mm radially, with the concave surface facing the stem center. The inner bundle tends to divide into three portions, with the abaxial surface of two bundles possessing protoxylem strands (Figure 5f, arrows). The two bundles in oblique longitudinal section may present the same shape and structure when they are in transverse view. Therefore, in the upward and outward course through the cortex of the stem, a leaf trace dichotomizes to form four bundles that assume two C-configurations in the petiole base. The tracheids of the vascular bundles are 10–52 μm in diameter and embedded in a ground tissue of parenchyma cells which are 30–110 μm in diameter. Bands of sclerenchyma cells occur in partially preserved sparganum-type outer cortex, and are 450–720 μm and 130–190 μm in radial and tangential dimensions, respectively (Figure 5b). Intervals between adjacent bands are ca. 260 μm.

### Systematics

? Class Spermatopsida

Order Incertae sedis

Genus *Yiduxylon* Wang et Liu gen. nov.

### Generic diagnosis

Stems with fronds borne helically in a 1/3 phyllotaxy. Frond rachises bifurcate proximally. Two orders of pinnae with alternately arranged secondary pinna rachises bearing alternate pinnules which are planate and highly dissected. Stems protostelic with small three-ribbed primary xylem, broad secondary xylem, and narrow cortex. Primary xylem mesarch with each rib possessing a single peripheral protoxylem strand. Secondary xylem intermediate between manoxylic and pycnoxylic types. Multiseriate bordered pits on tangential and radial walls of secondary xylem tracheids. Rays usually biseriate and very high. Cortex consisting of an outer sparganum zone and an inner parenchymatous ground tissue. Two leaf traces successively derived tangentially from tip of each primary xylem rib and once divided to produce four traces in stem cortex. Leaf traces possessing mesarch primary xylem. Petiole base containing vascular supply of four bundles in two C-shaped groups (each group with concave surface facing stem center and protoxylem strands on abaxial surface), peripheral and parallel bands of sclerenchyma cells, and ground tissue of parenchyma cells.

Type species *Yiduxylon trilobum* Wang et Liu sp. nov.

### Specific diagnosis

As in generic diagnosis. Stems 7.2-20 mm in diameter and attached by fronds at 45-60°. Frond rachises 5.2-10 mm and 4.8-6.3 mm in diameter before and after proximal bifurcation at about 60°, respectively. Narrow and longitudinally parallel superficial ridges and grooves alternating on stems and rachises of fronds and primary pinnae. Primary pinna rachises 1.8-2.6 mm in diameter. Secondary pinna borne at about 90°, with rachises 0.8-1.3 mm in diameter, and bearing pinnules at 45-90°. Pinnules up to 21.7 mm long and about 19.4 mm wide, with each unequally dividing into four or more units that are anisotomous twice or thrice. Ratio of primary xylem diameter to stem diameter 1/5. Stem primary xylem 1.8-2.0 mm in diameter, with three undivided ribs varying in length and becoming narrower towards stelar center. Secondary xylem up to 5.2 mm thick. Rays 2–6 cells wide and more than 120 cells high. Cortex 0.8-1.1 mm thick and inner cortex lacking sclerotic clusters. Individual leaf traces circular or slightly oval with two protoxylem strands. At petiole base, each C-shaped group of two vascular bundles asymmetrical with inner bundle tending to divide into three portions.

### Holotype designated here

Slide HBY-02 (Figure 2a, c-f, Additional file 2: Figure S2b and Additional file 3: Figure S3b).

### Paratypes designated here

Specimens PKUB14401 (Figure 1a), PKUB14403 (Figure 1b), PKUB14402a (Figure 1c), PKUB14402b (Figure 1d, e); Slides HBY-04 (Additional file 2: Figure S2c, f, h), HY3-4 (Figures 2b, 3d-f, Additional file 3: Figure S3g), HY9-2 (Figure 4e), 4–6 (Figure 5b, d-f).

### Etymology

Generic name from Yidu (where the plant was discovered) and the Greek xylon (xylem). Specific epithet from the Greek treis (tri) and lobos (lobes), meaning three-lobed shape of stem primary vascular system in transverse section.

### Locality and horizon

Tizikou, Maohutang village, Wangjiafan Town, Yidu City, Hubei Province, China; Upper Devonian (Famennian) Tizikou Formation.

### Repository

All specimens and slides are housed in the Department of Geology, Peking University, Beijing, China.

### Comparisons

Although the aneurophyte progymnosperms possess protostele with ribbed primary xylem of mesarch maturation, and secondary xylem with bordered pits on all walls of tracheids [1], they are distinct from *Yiduxylon* mainly in the geological age, other stelar architectures and vegetative morphology. Aneurophytes existed in the Middle Devonian-Late Devonian (Frasnian) and was characterized by protoxylem strands near the tips and along the midplanes of the primary xylem ribs and in the stelar center, pycnoxylic secondary xylem and radial divergence of branch traces [1,36,37]. Furthermore, except for *Triloboxylon ashlandicum* and *Proteokalon petryi* [38,39], the aneurophytes lack planation of the lateral axes (alternate or opposite arrangement) and vegetative ultimate appendages (planate leaves); a decussate pattern occurs in *Tetraxylopteris* with most orders of branches containing a four-ribbed primary vascular system [36]. Protoxylem strands along the midplanes of the rib of the Aneurophytes and Stenokoleales usually accompany and probably have been derived from a single strand at the stelar center [36,40,41]. Concerning *Yiduxylon*, the primary xylem is poorly preserved, but many sections indicate the absence of protoxylem strands along stelar ribs and two sections perhaps lack a central protoxylem strand (Additional file 4: Figure S4a, b). The secondary xylem is intermediate between pycnoxylic and manoxylic type (see below). Two leaf traces depart successively from the tip of a stem xylem rib (Additional file 6: Figure S6). One trace diverges and extends up at the boundary between secondary xylem and cortex, and then another trace is initiated. Two leaf traces probably divide tangentially from the rib tip.

Stenokoleales of the Middle Devonian to Early Carboniferous represents a distinct order within the “radiate protoxylem” group comprising plants such as aneurophytes and some early spermatophytes [41]. This order may have a close affinity with the seed plants [42]. Stenokoleales includes *Stenokoleos* and *Crossia* known only from anatomy [41,42] and is characterized by a three-ribbed protostele with a single protoxylem strand at the stelar center and numerous protoxylem strands along the midplanes of the primary xylem ribs. Secondary xylem is sometimes present but only in small amounts. In *Stenokoleos*, successive pairs of traces are produced from the opposite xylem ribs of stem, suggesting that paired fronds are borne alternately [43]. As to *Crossia*, three stelar ribs of the lower orders of axes divide to form six ribs where branch traces are emitted in pairs [41]. However, *Yiduxylon* has protoxylem strands restricted near the periphery of three xylem ribs of stem, large amounts of secondary xylem, two leaf traces successively arising from a xylem rib, and helically arranged petioles.

The stem steles of early seed plants (Late Devonian to Carboniferous Buteoxylonales, Calamopityales and Lyginopteridales of seed ferns) may be divided into three types, i.e. ribbed protostele or actinostele, parenchymatized protostele and eustele with pith [37]. *Yiduxylon* with protostele differs from the spermatophytes with parenchymatized protosteles, e.g., *Buteoxylon* [44], *Galtiera* [15], *Heterangium* [45,46], *Rhetinangium* [47-49], *Schopfiastrum* [50], *Microspermopteris* [51,52], some species of *Calamopitys*, e.g., *C. saturni* and *C. annularis* [53], and most species of *Stenomyelon*, e.g., *S. muratum* [11], *S. bifasciculare* [54], *S. tuedianum* [55,56]. *Yiduxylon* also differs from those with eusteles, e.g., *Diichnia* [11,12,16], *Faironia* [4], *Lyginopitys* [57], *Lyginopteris* [58], *Trivena* [20], *Triichnia* [59], and some species of *Calamopitys*, e.g., *C. solmsii* [53].

The spermatophytes with ribbed protosteles include *Bostonia* [60,61], *Elkinsia* [10], *Kerryoxylon* [7], *Laceya* [8,9], *Stenomyelon primaevum* [55,56], *Tetrastichia* [7,62,63], *Triradioxylon* [64], and *Tristichia* [7,65,66]. However, *Tetrastichia* and *Kerryoxylon* differ from *Yiduxylon* in having four- and six-ribbed primary xylem, respectively. The primary xylem of *Tetrastichia* is sometimes three- or five-ribbed, and a single protoxylem strand is present near the stelar center, or along the midplane of each primary xylem rib; the phyllotaxy of fronds is opposite decussate, 1/3 and 2/5. Leaf traces of *Kerryoxylon* are three-ribbed in the stem cortex.

*Yiduxylon* resembles *Bostonia*, *Elkinsia*, *Laceya*, *Stenomyelon primaevum*, *Triradioxylon* and *Tristichia* in the protosteles with three primary xylem ribs possessing peripheral protoxylem strands. Nevertheless, *Yiduxylon* is characterized by two leaf traces successively derived in tangential pattern from a single rib of stem primary xylem. Specifically, the primary xylem of *Bostonia* comprises three semi-discrete portions and a central portion; the protoxylem strands occur also in the stelar center; neither xylem ribs nor peripheral protoxylem strands are longitudinally continuous; the vascular supply of petioles is three-ribbed and derived from a pair of leaf traces [61]. Protoxylem strands of *Elkinsia* are located also in the stelar center and sometimes along the midplane of xylem ribs; the peripheral protoxylem strand diverges into different numbers and shapes of leaf traces (one papillionoid, one C-shaped, two bilobed, four elliptical ones), although the frond rachis is vascularized by two C-shaped bundles [10]. The leaf trace initiation of *Laceya* is caused by repeated divisions of primary xylem rib resulting in a tip with 6–8 united lobes; the U-shaped leaf trace remains undivided and its abaxial surface shows 6–8 protoxylem strands [8]; vascular supply at the petiole base is a U-/V-shaped bundle with 8–12 protoxylem strands on the convex abaxial surface [9]. The primary xylem of *Stenomyelon primaevum* is separated from the secondary xylem by parenchymatous cells; protoxylem strands occur along the midplanes of xylem ribs and are often associated with a lacuna; pits appear only on radial walls of secondary xylem tracheids; there are eight vascular bundles in each petiole base [56]. One or more protoxylem strands are also located in the stelar center of *Triradioxylon*, and the leaf traces are undivided and the vascular supply in the petiole is T-shaped [64]. Concerning *Tristichia*, protoxylem strands sometimes occur also in the stelar center and along the xylem ribs, and the leaf traces are tetrarch and papillionoid [66]. Sclerotic nests characteristic of the inner cortex of *Elkinsia*, *Triradioxylon* and *Tristichia* are absent in *Yiduxylon*.

As in *Yiduxylon*, the pinnae and pinnules of Late Devonian *Kongshania* [3] are alternately arranged. In contrast, the pinnules of *Kongshania* are laminate and wedge-tongue shaped; the poorly preserved frond rachis in description and diagram consists mainly of two bean-shaped vascular bundles surrounded by secondary xylem, which are however difficult to see in the photographs.

## Discussion

### Taxonomy

It is widely agreed that the seed plants originated from the aneurophyte progymnosperms [1]. As Galtier [37] summarized, the early spermatophytes are characterized by both primitive aneurophyte and derived morphological and anatomical features. Primitive anatomical features include the protostelic primary vascular system, secondary xylem/phloem produced by a bifacial cambium, multiseriate and elliptical bordered pitting of tracheids, and outer cortex with longitudinally oriented plates of sclerenchyma cells. Derived features refer to such as bipartite fronds with dichotomous rachis, dorsiventral (adaxial/abaxial) identity of petioles, nonlaminate to entire pinnules, loss of protoxylem strands in stelar center, pits restricted to the radial walls of secondary xylem tracheids, and tangential divergence of leaf traces. In addition, the early seed plants often have their fronds borne helically on the stem [67]. *Yiduxylon* possesses these characteristic vegetative morphology and stelar architecture except for unknown secondary phloem. Manoxylic secondary xylem is another important feature of the early seed plants but it is difficult to define [37] or may be described as having large tracheids (more than 150 μm in diameter) and rays (over 5 cells wide and up to 200 cells high), which contrasts to pycnoxylic woods with small tracheids (less than 50 μm in diameter) and rays (uniseriate and short) [68,69]. In this case, the size of secondary xylem tracheids (12–75 μm in diameter) and rays (2–6 cells wide and more than 120 cells high) of *Yiduxylon* is intermediate between pycnoxylic and manoxylic wood anatomy.

The Buteoxylonales has only two genera, i.e., Early Carboniferous *Buteoxylon* [44] and *Triradioxylon* [64], with the former also reported from the Latest Devonian [6]. Calamopityales diversified in the Early Carboniferous and Lyginopteridales in the Carboniferous, but they (probably) occurred in the Late Devonian [1,20]. As noted by many workers [4,15,20,37,67], the Calamopityales and Lyginopteridales are difficult to define in that they share many characters such as helical phyllotaxis, protostele to eustele, cortex with sclerotic clusters and sclerenchyma cells (*sparganum* and *dictyoxylon* type). Their differentiation may depend on a combination of morphological (e.g., adventitious roots) and anatomical characters such as the ratio of primary vasculature diameter to stem diameter, leaf trace origination and vascular supply of frond rachises. Among them, the type of petiole vascular supply is crucial to define the Calamopityales and Lyginopteridales [64], although this character has been questioned (especially when it is independently used) because of considerable overlap of petiole morphology between the two orders [4,15,20].

According to Galtier [37] and Dunn et al. [20], adventitious roots are generally absent in the Calamopityales, the ratio of primary vasculature diameter to stem diameter is 1/5-1/10, the leaf trace origination involves multiple divisions and sometimes is double, the petiole is *Kalymma* type with four or more vascular bundles in the base of a frond rachis (multifascicular petiole), the total volume of these bundles exceeds the volume of cauline primary vasculature at the same level. The cortex of the calamopityaleans has been emphasized as remarkably broad [1,70]. In contrast, the Lyginopteridales is characterized by adventitious roots, the ratio of primary vasculature diameter to stem diameter is 1/2-1/4, the leaf trace usually remains undivided to produce a single petiolar vascular supply with several protoxylem strands, or it divides once into two closely associated bundles of U-, or V-, or W-shape (*Lyginorachis*-type petiole), the volume of the petiolar vascular supply never exceeds the stelar volume. There are however some exceptions to the characters of Lyginopteridales. For example, *Laceya* probably has no adventitious roots [9] and *Heterangium* may possess *Kalymma*-type petioles [71].

*Yiduxylon* cannot be assigned to the Buteoxylonales with their undivided leaf trace and T-shaped petiolar vascular supply. It differs from the Elkinsiales represented by *Elkinsia* mainly in the stelar architecture and leaf trace number/configurations (see Comparisons). *Yiduxylon* lacks adventitious roots, has a ratio of stelar diameter to stem diameter of 1/5, double leaf traces in origin, four leaf traces in the inner cortex, and a multifascicular petiole base with four discrete bundles that tend to further divide and exceed the volume of stem primary vasculature. These characters suggest anatomical similarities with the Calamopityales. However, the ratio of 1/5 is not a very low value and it results mainly from the small primary xylem and thick secondary xylem (i.e., the cortex is thin). Individual leaf traces are undivided in the cauline secondary tissue. Furthermore, as in some lyginopteridaleans, the four petiolar bundles of *Yiduxylon* are in two C-shaped traces that possess protoxylem strands on the abaxial surface. The origin of the leaf traces is unique because the two traces were produced from a stem primary xylem rib at different stages. *Yiduxylon* further exemplifies the anatomical similarities among early seed plants and indicates that their taxonomy depends primarily on fertile organs with attachment or association of stem/frond anatomy.

### Growth habit

Stems of the Lyginopteridales and Calamopityales are generally less and more than 20 mm in diameter, respectively [37]. As summarized by Dunn et al. [20], due to the presence of adventitious roots, narrow stems and large fronds, the lyginopteridaleans are probably more similar to lianas or vines and possess a scrambling or climbing habit; the calamopityaleans lacking adventitious roots are considered to be primarily upright plants. In addition, the wide angle of attachment of the fronds and *Dictyoxylon*-type outer cortex of the lyginopteridaleans are thought to be adaptations to the liana habit [58]. Adventitious roots are absent in the specimens and stem sections of *Yiduxylon*, the outer cortex is *Sparganum*-type, and the angle of frond attachment is not so wide, whereas the stem diameter is generally less than 20 mm and the complex fronds consist of two orders of long pinnae with many large pinnules. Therefore, it is difficult to decide the growth habit of *Yiduxylon*, although it appears to have had an upright stem. Future biomechanical analyses such as those by Masselter et al. [21] may provide more accurate information on this subject.

### Evolution of vegetative organs and protostele in seed plants

The aneurophyte progymnosperms of Middle to Late Devonian (Givetian-Frasnian) were widespread on the Laurussia continent and Gondwana continents. As the descendant of the aneurophytes, the spermatophytes were widespread in the Late Devonian (Famennian) of Laurussia. However, surprisingly, the aneurophytes have not been reported from South China [72,73]. There are now three genera of seed plants known from the Famennian of China or eastern Asia, i.e., *Cosmosperma* [2], *Kongshania* [3] and *Yiduxylon* (this paper). In *Yiduxylon*, the secondary pinnae and highly dissected pinnules are borne alternately. These vegetative characters support the hypothesis that Late Devonian seed plants generally possess pinnae in one plane and planate rather than laminate pinnules, in contrast to the three-dimensional branches and vegetative ultimate appendages of the aneurophytes [2].

Various stelar configurations occur in the early seed plants, ranging from simple protostele to eustele with sympodia and pith. The eustele is suggested to have evolved from the protostele (e.g., *Stenomyelon primaevum*) by gradual loss of the tracheidal tissue in the center of protostele; the transitional stelar form refers to parenchymatized protostele (e.g., *Stenomyelon tuedianum* and some species of *Calamopitys*), in which the stele has both parenchyma cells and tracheids [1,22,23,74]. Parenchymatized protostele and eustele were widespread in the Carboniferous Buteoxylonales, Calamopityales and Lyginopteridales.

Except for *Buteoxylon gordonianum* [6], where the stem primary vascular system with pith is unclear in detail, Late Devonian (Famennian) spermatophytes are primitive in having a protostele with three to six ribs. These plants are *Elkinsia polymorpha* [10], *Kerryoxylon hexalobatum* [7], *Laceya hibernica* [8,9], *Tristichia* sp. [7], cf. *Tetrastichia bupatides* [7] and *Yiduxylon trilobum* (this paper). Among them, *Elkinsia polymorpha* possesses a three-ribbed primary xylem with a single central protoxylem strand producing other strands along the ribs, which is very similar to the condition of the ancestral aneurophytes. Its frond rachises contain a *Lyginorachis*-like trace with abaxial protoxylem strands, which characterizes the early spermatophytes. This combination of stelar and petiolar architectures indicates the close evolutionary relationship between the aneurophytes and spermatophytes [75]. Therefore, middle Famennian *Elkinsia* with its protostele is more primitive than other Famennian taxa. In the Latest Devonian seed plants from Ireland, the protoxylem strand occurs in/near the stelar center of *Tristichia* sp. and cf. *Tetrastichia bupatides*, or along the midplane of stelar ribs of *Laceya hibernica*, or only at the periphery of xylem rib of *Kerryoxylon hexalobatum. Yiduxylon* is probably younger than *Elkinsia* but older than the Irish taxa. If *Yiduxylon* had only peripheral protoxylem strands, it suggests that the protostele of early seed plants may have lost the protoxylem strands in the stelar center and along the stelar ribs by the Famennian. Ribbed protostele extends to the earliest Carboniferous (late Tournaisian) *Triradioxylon primaevum* [64], species of *Tristichia* [66] and *Bostonia perplexa* [61] with central protoxylem strands, *Stenomyelon primaevum* [55,56] and *Tetrastichia bupatides* [62,63] without such strands.

**Figure 6:**
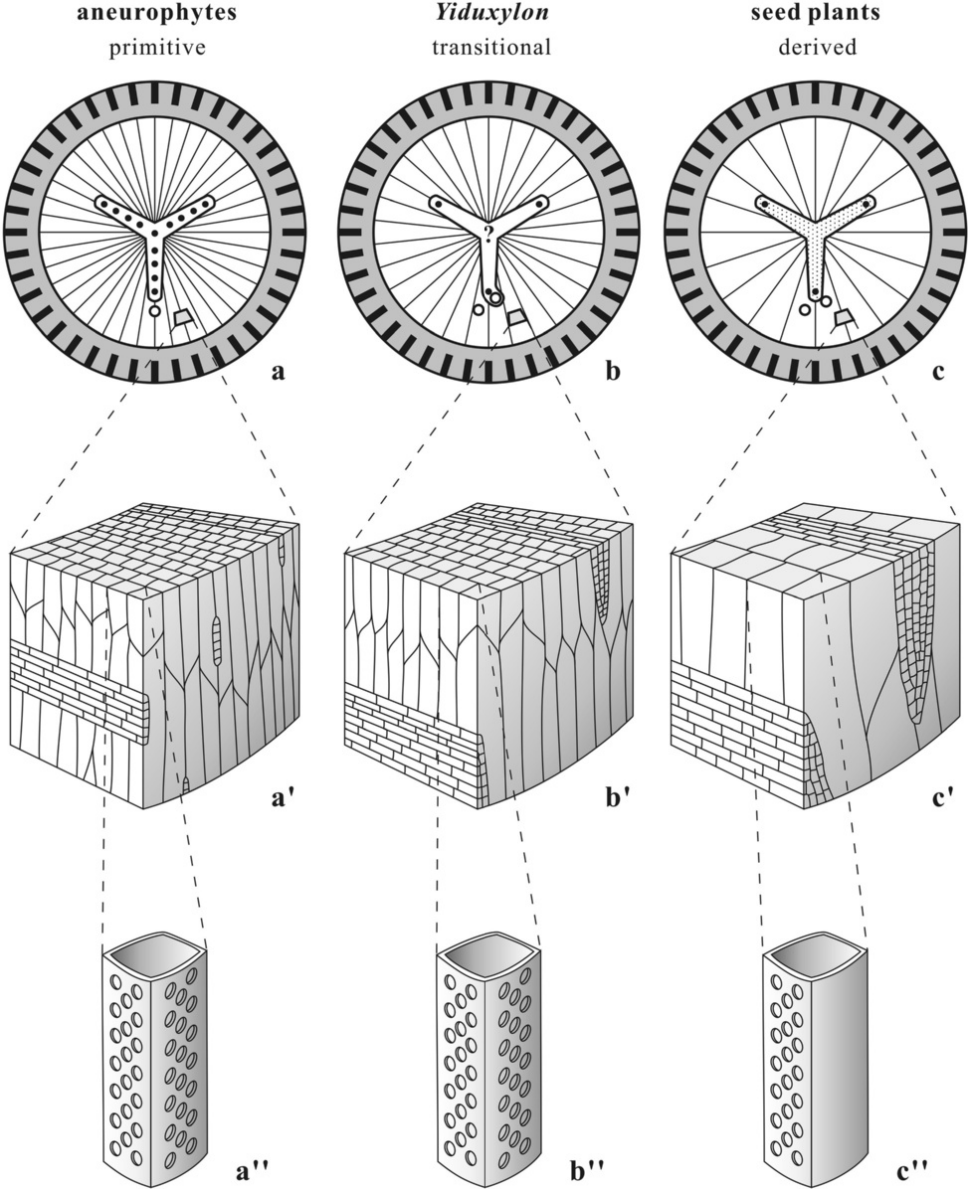
**Diagram showing the transitional traits of stelar structure and leaf trace formation of** ***Yiduxylon trilobum.*** **(a-c)** Transverse section of stem showing three-ribbed primary xylem, broad secondary xylem (represented by thin lines directed radially), narrow cortex with outer cortex containing peripheral bands of sclerenchyma cells (thick lines directed radially). **(a’-c’)** Three-dimensional view of secondary xylem with tracheids (vertically aligned) and ray parenchyma cells (horizontally aligned). **(a“-c”)** Secondary xylem tracheid with bordered pits. **(a-a’)** Aneurophyte progymnosperms showing protostele with a central and several protoxylem strands (black dots) along primary xylem rib, radial divergence of branch trace (small circle) **(a)**, pycnoxylic secondary xylem with small tracheids and rays **(a')**, and pits on both radial and tangential walls of tracheid **(a")**. **(b-b“)** *Yiduxylon* with protostele possessing a protoxylem strand near the periphery of primary xylem rib but lacking strands along rib and ? a central strand, tangential divergence of leaf traces (small circles) **(b)**, pycnoxylic-manoxylic secondary xylem with moderately sized tracheids and rays **(b')**, and pits on both radial and tangential walls of tracheid **(b“)**. **(c-c”)** Early seed plants with parenchymatized protostele possessing a protoxylem strand near periphery of primary xylem rib, tangential divergence of leaf traces **(c)**, manoxylic secondary xylem with large tracheids and rays **(c’)**, and pits restricted on radial walls of tracheid **(c”)**.

It is clear that the protosteles of *Elkinsia* [10], *Laceya* [8] and *Yiduxylon* are not fully evolved, as they still show the aneurophyte character of bordered pits on both radial and tangential walls of secondary xylem tracheids. This feature also occurs in *Tetrastichia* [62], *Triradioxylon* [64] and the earliest Carboniferous (Tournaisian) *Stenomyelon bifasciculare* with parenchymatized protosteles [54]. Secondary xylem of *Elkinsia* [10] may be pycnoxylic in that the tracheids are small (15–88 μm in diameter) and rays are 1–2 cells wide. That of *Buteoxylon* [6] and *Laceya* [8] is manoxylic. Secondary xylem of *Yiduxylon* is probably intermediate between pycnoxylic and manoxylic types separately characterizing the aneurophytes and spermatophytes (see above). However, *Elkinsia*, *Laceya* and *Yiduxylon* demonstrate the derived features of bipartite fronds with dorsiventral identity and tangential divergence of leaf traces. In general, the Late Devonian and some of the earliest Carboniferous seed plants possessed the more or less protostelic architectures of ancestral progymnosperms. Among these taxa, *Yiduxylon* obviously demonstrates the transitional stelar traits between these two plant groups. Such traits include primary vascular system, type of secondary xylem, pit distribution on secondary xylem tracheid walls, and mode of leaf trace production (Figure 6).

### Palaeogeographic consideration

It is commonly accepted that the origin of seed plants is closely related to the aneurophyte gymnosperms. Accordingly, *Yiduxylon* provides transitional anatomical evidence linking these two plant groups. However, as stated before in this paper, typical aneurophytes have not yet been recognized from South China and thus need to be sought after in the Middle Devonian (Givetian) flora of this block. Alternatively, the seed plants may be nested within earlier representatives, e.g., *Celatheca beckii* with fertile structures putatively resembling cupulate ovules or pollen organs, which occurs in the Lower Devonian (Pragian) Posongchong Formation, Yunnan Province in South China [76-78].

At present, most of the earliest seed plants have been discovered from the Late Devonian of the Laurussian continent. Other coeval spermatophyte taxa come from South China which was an isolated block near the equator during the Devonian [79,80]. The Late Devonian flora of South China is well known for other lineages of vascular plants including many cosmopolitan and/or endemic elements. These lineages include the lycopsids, preferns, sphenophyllalean sphenopsids and archaeopterid progymnosperms. The rare record of Famennian spermatophytes in China may be related to the palaeogeographic position, or palaeoclimatic influence, limited dispersal, or insufficient collection in the past.

## Conclusions

*Yiduxylon* is the third genus of seed plants or a taxon closely related to seed plants to have been reported from the Late Devonian of China or East Asia. *Yiduxylon* provides evidence for stem/petiole anatomy in this period. Its vegetative organs support the hypothesis that Late Devonian spermatophytes had evolved planate pinnae and pinnules. *Yiduxylon*, probably upright in habit, differs from other early seed plants mainly in the stem protostele with three-ribbed primary xylem of mesarch maturation, successive divergence of two leaf traces in tangential pattern from a primary xylem rib, four leaf traces in the cortex, and a petiolar base with four vascular bundles in two C-shaped groups. The difficulty in differentiating stelar architecture of the early seed plants indicates that they are anatomically similar and their taxonomy depends largely on fertile organs. The stems of all Late Devonian and some of the earliest Carboniferous seed plants possessed a ribbed protostele, which lacks or contains a central protoxylem strand as a character of the ancestral aneurophyte progymnosperms. Protostelic architecture and leaf trace formation of *Yiduxylon* suggest transitional traits in the evolution of early seed plants from aneurophytes.

## Additional files

Additional file 1: Figure S1.
*Yiduxylon trilobum* gen. et sp. nov. from Hubei. **(a-b)** Line-drawings of part and counterpart of specimen in Figure 1c,d, respectively. fr: frond rachis, ppr: primary pinna rachis, spr: secondary pinna rachis, p: pinnule. PKUB14402a, PKUB14402b. Additional file 2: Figure S2. Transverse sections of a stem of *Yiduxylon trilobum* gen. et sp. nov. from Hubei. **(a-d)** Successive transverse sections of a stem showing small three-ribbed primary xylem, broad secondary xylem, and narrow sparganum-type cortex. HBY-01, HBY-02, HBY-04, HBY-16. **(a)** Arrow indicating part enlarged in **(e)**. **(c)** Left and right arrows indicating parts enlarged in **(f)** and **(h)**, respectively. **(d)** Upper and lower arrows indicating parts enlarged in **(g)** and **(i)**, respectively. **(e-g)** Individual leaf traces in inner cortex. Enlargement of (**a**, arrow), (**c**, left arrow) and (**d**, upper arrow), respectively. **(h)** Enlargement of (**c**, right arrow) showing leaf trace diverging from tip of a primary xylem rib. **(i)** Earlier stage of leaf trace divergence in **(h)**. HBY-03. **(j)** Enlargement of (**d**, lower arrow) showing leaf trace in secondary xylem. Arrows indicating two protoxylem strands. HBY-16. **(k)** Earlier stage of leaf trace divergence in **(j)**. Arrow showing a protoxylem strand near rib tip of stem primary xylem. HBY-11. Scale bars = 0.2 mm **(e-k)**. Additional file 3: Figure S3. Transverse sections of stems of *Yiduxylon trilobum* gen. et sp. nov. from Hubei. **(a-b)** Enlargement of Additional file 2: Figure S2a,b, respectively. Three-ribbed primary xylem surrounded by secondary xylem. Arrows 1–3 in **(a)** showing three xylem ribs, with arrows 2–3 also indicating parts enlarged in **(c-d)**, respectively. **(c)** Enlargement of (**a**, arrow 2) showing protoxylem strand (arrow). **(d)** Enlargement of (**a**, arrow 3) showing protoxylem strand (left arrow) and incipient leaf trace (right arrow). **(e-g)** Successive transverse sections of a stem showing three-ribbed primary xylem of mesarch maturation. HY3-1, HY3-3, HY3-4. Arrows in **(e-f)** indicating parts enlarged in **(h)** and **(i)**, respectively. **(g)** Enlargement of Figure 2b. **(h-i)** Enlargement of (**e**, arrow) and (**f**, arrow), respectively, showing peripheral protoxylem strand. Scale bars = 0.2 mm **(c, d, h, i)** , 0.5 mm **(a, b, e-g)**. Additional file 4: Figure S4. Transverse sections of stems of *Yiduxylon trilobum* gen. et sp. nov. from Hubei. **(a)** Enlargement of Additional file 3: Figure S3e showing primary xylem probably without central protoxylem strand. Arrow indicating a protoxylem strand near apex of xylem tip. **(b)** Three-ribbed stem primary xylem probably lacking central protoxylem strand but with peripheral strands near rib tip. 2–1. Scale bars = 0.2 mm. Additional file 5: Figure S5. Transverse sections of a stem of *Yiduxylon trilobum* gen. et sp. nov. from Hubei. **(a-g)** Serial sections showing stages of two leaf traces. 3–1 to 3–7. Arrows in **(f-g)** indicating parts enlarged in **(h)** and **(i)**, respectively. **(h)** Enlargement of (**f**, arrow) showing leaf trace in secondary xylem and its protoxylem strand (arrow). **(i)** Enlargement of (**g**, arrow) showing leaf trace at periphery of inner cortex and two protoxylem strands (arrows). Scale bars = 0.2 mm **(h, i)**, 0.5 mm **(a-g)**. Additional file 6: Figure S6.
*Yiduxylon trilobum* gen. et sp. nov. from Hubei. **(a-g)** Line-drawings of serial transverse sections of a stem in Additional file 5: Figure S5a-g, respectively. 3–1 to 3–7. **(h-i)** Line-drawings of portions of two transverse sections of a stem in Additional file 2: Figure S2a,c, respectively. HBY-01, HBY-04. white area: primary xylem of stem or leaf trace, small circle: protoxylem strand, oblique lines: secondary xylem, gray area: cortex.
