# Supplementary figures and images for: A new Late Devonian genus with seed plant affinities
Source: BMC Evol Biol. 2015 Feb 26;15:28. doi: 10.1186/s12862-015-0292-6 (PMC4341886; doi:10.1186/s12862-015-0292-6)

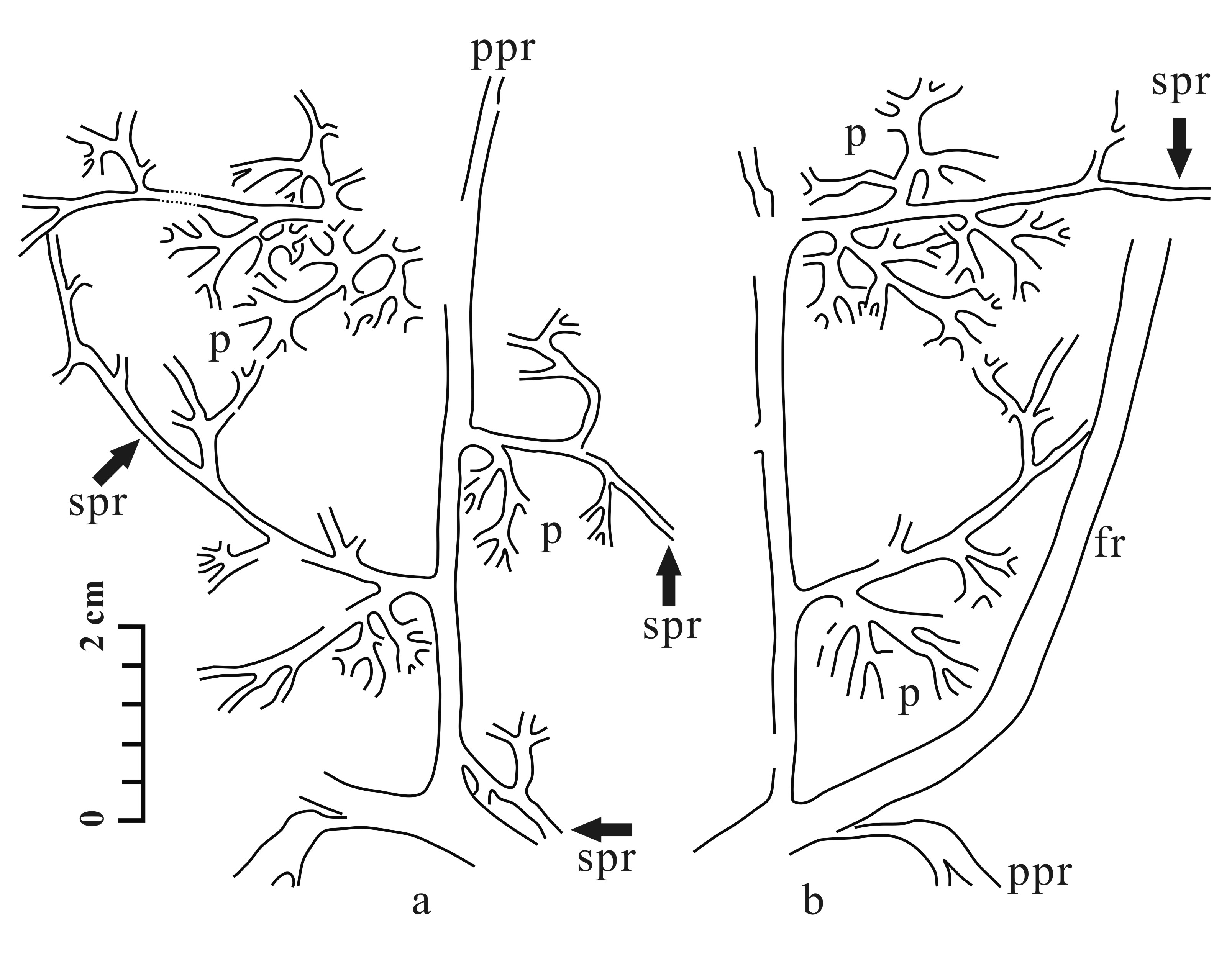

Supplement: Additional file 1: Figure S1. — Yiduxylon trilobum gen. et sp. nov. from Hubei. (a-b) Line-drawings of part and counterpart of specimen in Figure 1c,d, respectively. fr: frond rachis, ppr: primary pinna rachis, spr: secondary pinna rachis, p: pinnule. PKUB14402a, PKUB14402b. [file 12862_2015_292_MOESM1_ESM.jpeg]

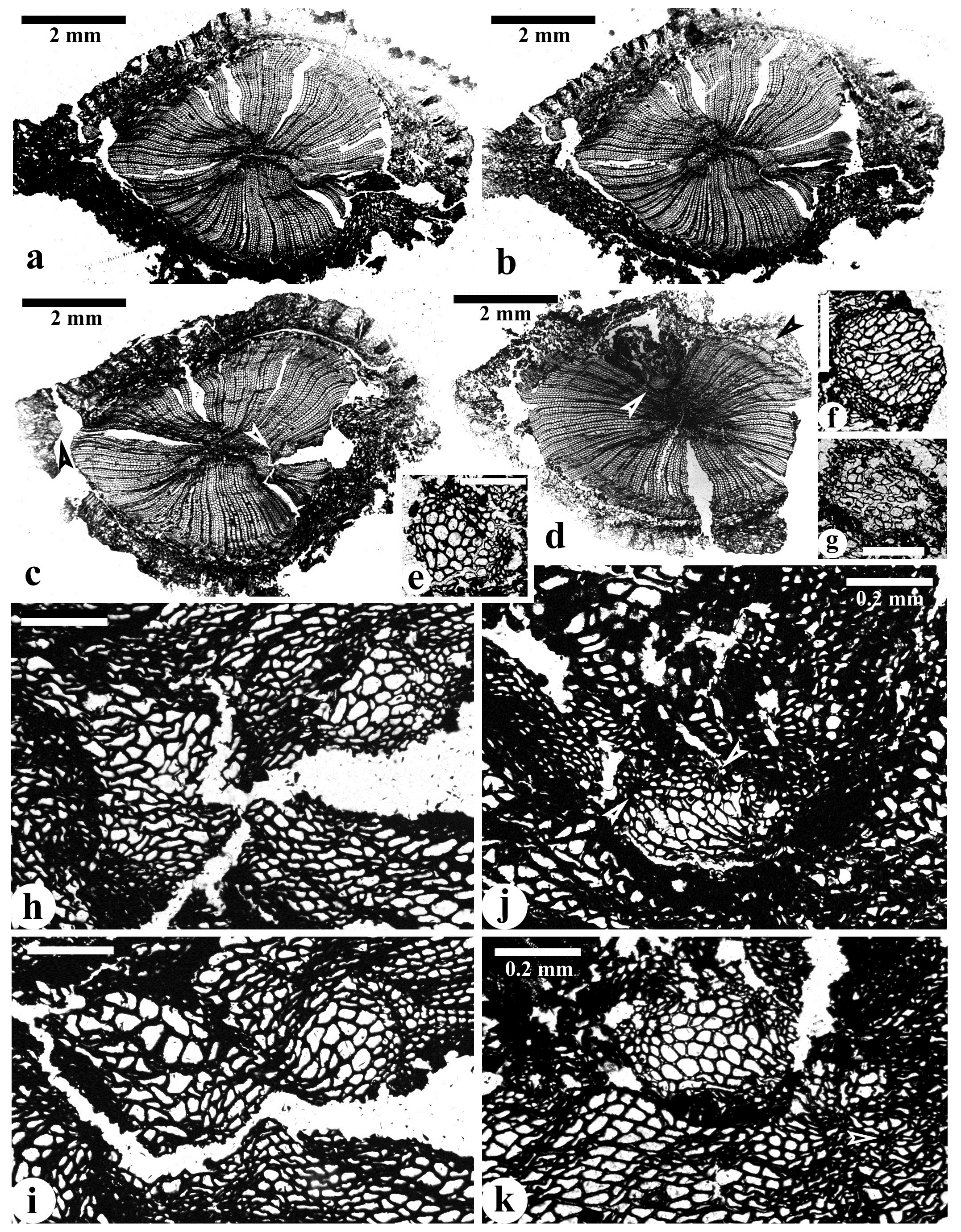

Supplement: Additional file 2: Figure S2. — Transverse sections of a stem of Yiduxylon trilobum gen. et sp. nov. from Hubei. (a-d) Successive transverse sections of a stem showing small three-ribbed primary xylem, broad secondary xylem, and narrow sparganum-type cortex. HBY-01, HBY-02, HBY-04, HBY-16. (a) Arrow indicating part enlarged in (e). (c) Left and right arrows indicating parts enlarged in (f) and (h), respectively. (d) Upper and lower arrows indicating parts enlarged in (g) and (i), respectively. (e-g) Individual leaf traces in inner cortex. Enlargement of (a, arrow), (c, left arrow) and (d, upper arrow), respectively. (h) Enlargement of (c, right arrow) showing leaf trace diverging from tip of a primary xylem rib. (i) Earlier stage of leaf trace divergence in (h). HBY-03. (j) Enlargement of (d, lower arrow) showing leaf trace in secondary xylem. Arrows indicating two protoxylem strands. HBY-16. (k) Earlier stage of leaf trace divergence in (j). Arrow showing a protoxylem strand near rib tip of stem primary xylem. HBY-11. Scale bars = 0.2 mm (e-k). [file 12862_2015_292_MOESM2_ESM.jpeg]

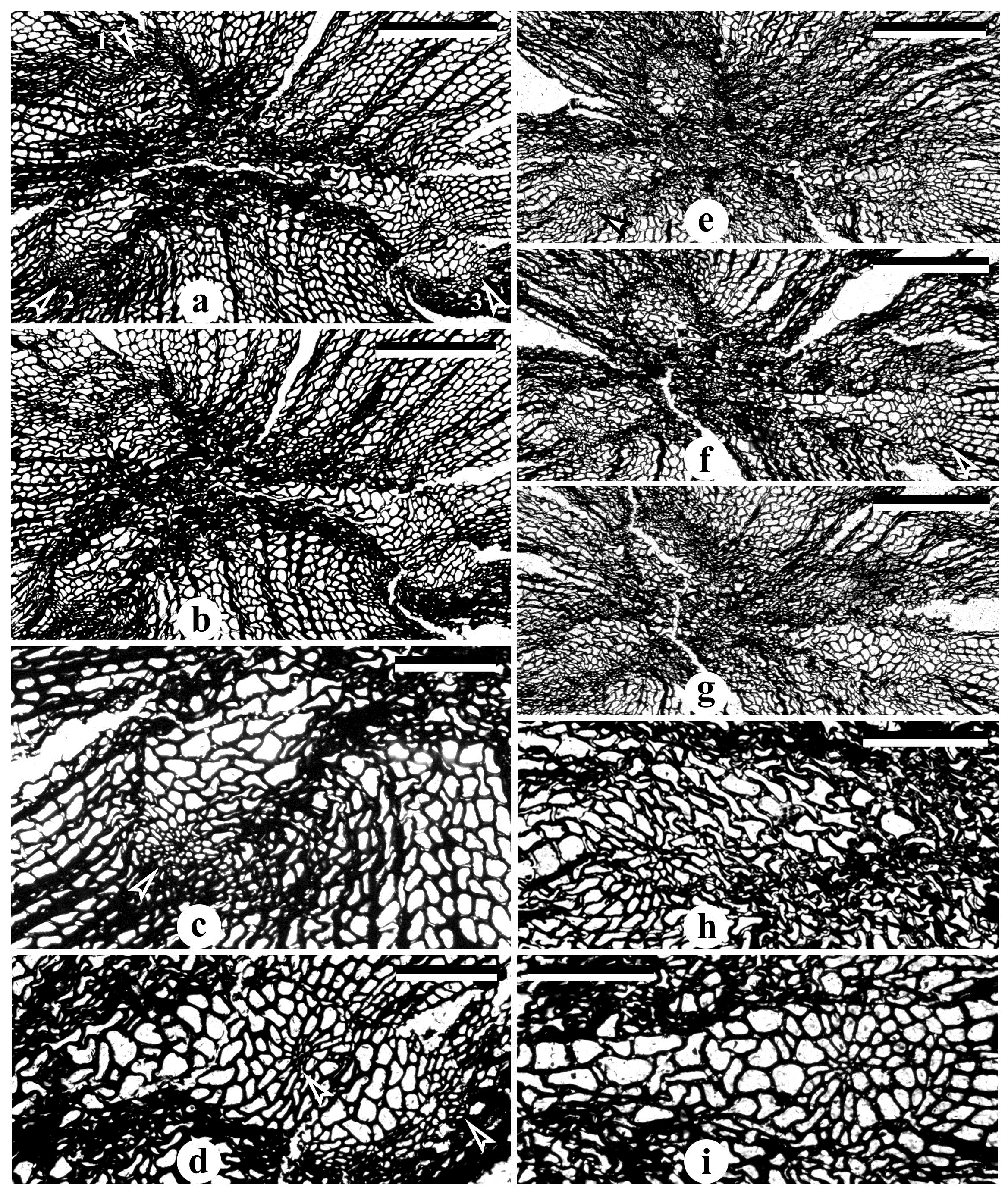

Supplement: Additional file 3: Figure S3. — Transverse sections of stems of Yiduxylon trilobum gen. et sp. nov. from Hubei. (a-b) Enlargement of Additional file 2: Figure S2a,b, respectively. Three-ribbed primary xylem surrounded by secondary xylem. Arrows 1–3 in (a) showing three xylem ribs, with arrows 2–3 also indicating parts enlarged in (c-d), respectively. (c) Enlargement of (a, arrow 2) showing protoxylem strand (arrow). (d) Enlargement of (a, arrow 3) showing protoxylem strand (left arrow) and incipient leaf trace (right arrow). (e-g) Successive transverse sections of a stem showing three-ribbed primary xylem of mesarch maturation. HY3-1, HY3-3, HY3-4. Arrows in (e-f) indicating parts enlarged in (h) and (i), respectively. (g) Enlargement of Figure 2b. (h-i) Enlargement of (e, arrow) and (f, arrow), respectively, showing peripheral protoxylem strand. Scale bars = 0.2 mm (c, d, h, i) , 0.5 mm (a, b, e-g). [file 12862_2015_292_MOESM3_ESM.jpeg]

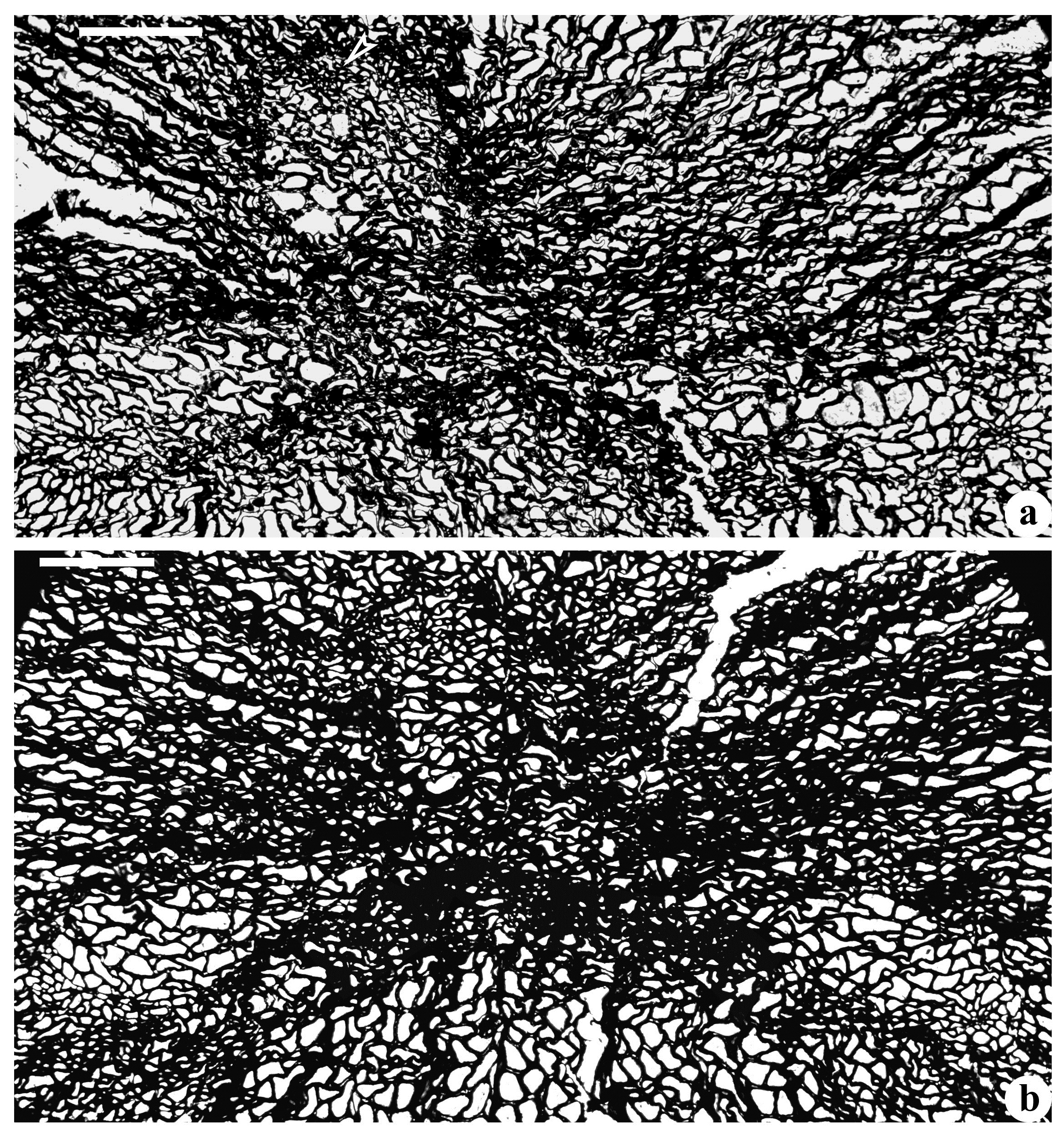

Supplement: Additional file 4: Figure S4. — Transverse sections of stems of Yiduxylon trilobum gen. et sp. nov. from Hubei. (a) Enlargement of Additional file 3: Figure S3e showing primary xylem probably without central protoxylem strand. Arrow indicating a protoxylem strand near apex of xylem tip. (b) Three-ribbed stem primary xylem probably lacking central protoxylem strand but with peripheral strands near rib tip. 2–1. Scale bars = 0.2 mm. [file 12862_2015_292_MOESM4_ESM.jpeg]

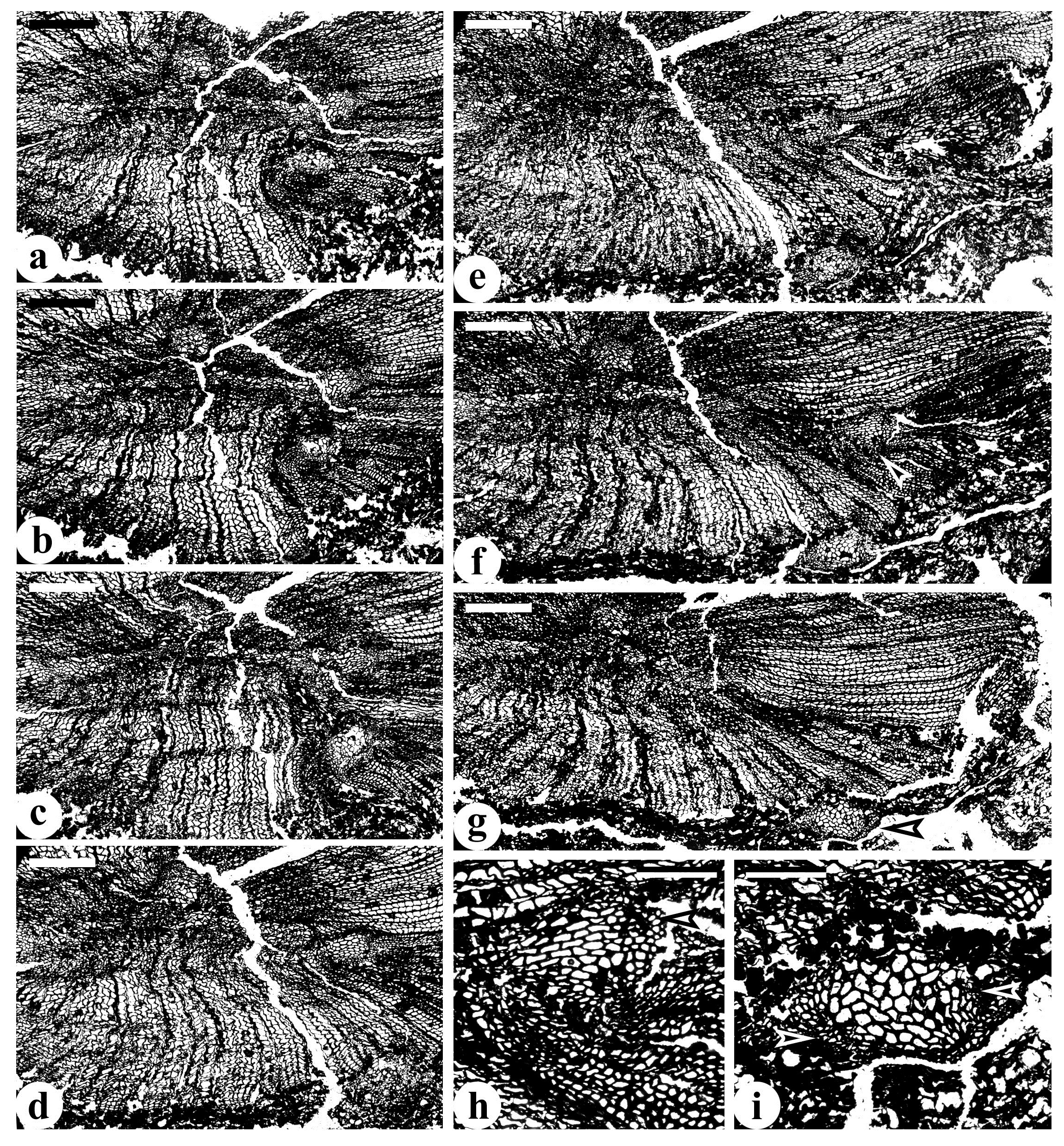

Supplement: Additional file 5: Figure S5. — Transverse sections of a stem of Yiduxylon trilobum gen. et sp. nov. from Hubei. (a-g) Serial sections showing stages of two leaf traces. 3–1 to 3–7. Arrows in (f-g) indicating parts enlarged in (h) and (i), respectively. (h) Enlargement of (f, arrow) showing leaf trace in secondary xylem and its protoxylem strand (arrow). (i) Enlargement of (g, arrow) showing leaf trace at periphery of inner cortex and two protoxylem strands (arrows). Scale bars = 0.2 mm (h, i), 0.5 mm (a-g). [file 12862_2015_292_MOESM5_ESM.jpeg]

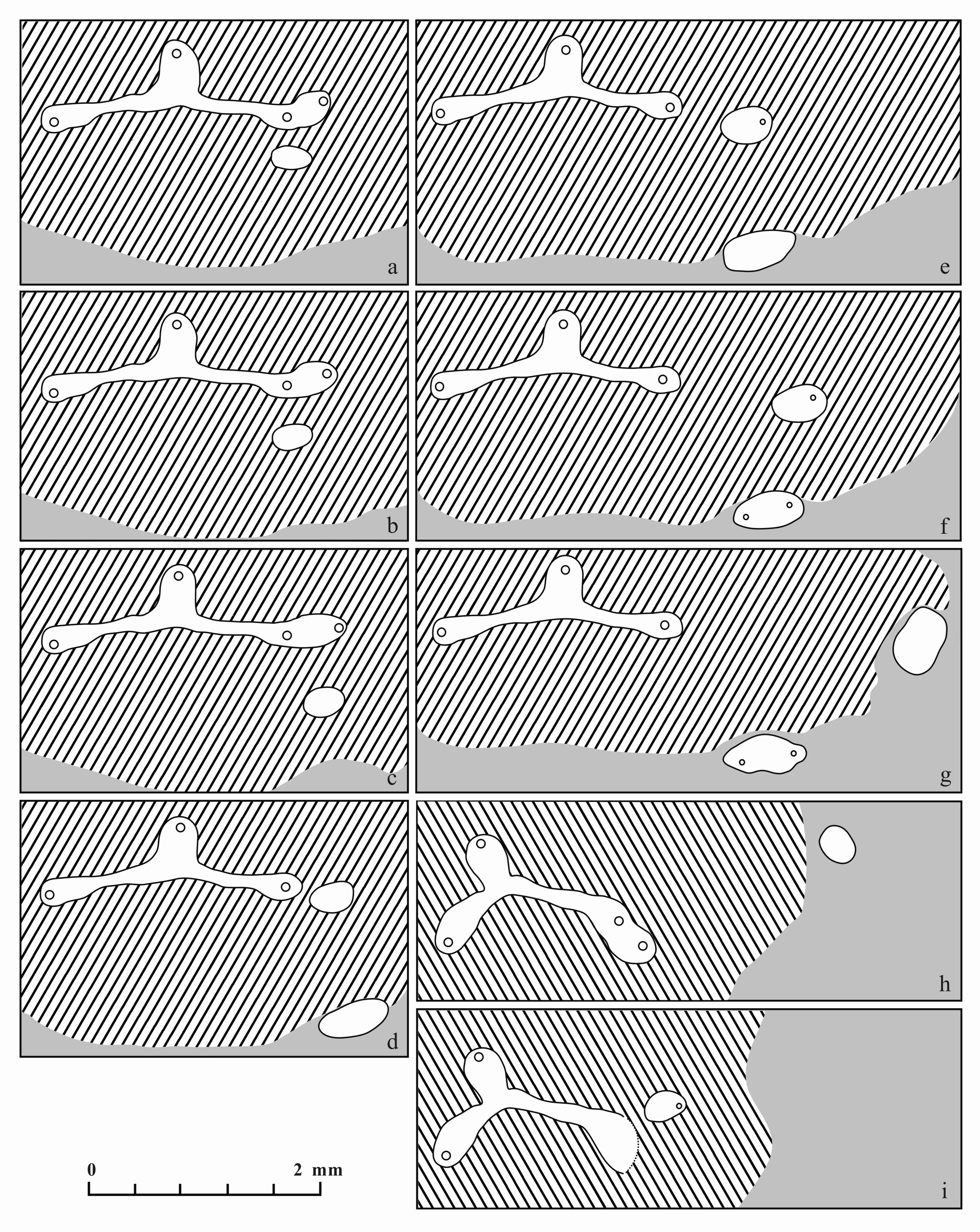

Supplement: Additional file 6: Figure S6. — Yiduxylon trilobum gen. et sp. nov. from Hubei. (a-g) Line-drawings of serial transverse sections of a stem in Additional file 5: Figure S5a-g, respectively. 3–1 to 3–7. (h-i) Line-drawings of portions of two transverse sections of a stem in Additional file 2: Figure S2a,c, respectively. HBY-01, HBY-04. white area: primary xylem of stem or leaf trace, small circle: protoxylem strand, oblique lines: secondary xylem, gray area: cortex. [file 12862_2015_292_MOESM6_ESM.jpeg]
